# Factors Associated with Variations in Population HIV Prevalence across West Africa: Findings from an Ecological Analysis

**DOI:** 10.1371/journal.pone.0142601

**Published:** 2015-12-23

**Authors:** Holly J. Prudden, Tara S. Beattie, Natalia Bobrova, Jasmina Panovska-Griffiths, Zindoga Mukandavire, Marelize Gorgens, David Wilson, Charlotte H. Watts

**Affiliations:** 1 Department of Global Health and Development, London School of Hygiene and Tropical Medicine, London, United Kingdom; 2 The World Bank, Washington, District of Columbia, United States of America; University of Missouri-Kansas City, UNITED STATES

## Abstract

**Background:**

Population HIV prevalence across West Africa varies substantially. We assess the national epidemiological and behavioural factors associated with this.

**Methods:**

National, urban and rural data on HIV prevalence, the percentage of younger (15–24) and older (25–49) women and men reporting multiple (2+) partners in the past year, HIV prevalence among female sex workers (FSWs), men who have bought sex in the past year (clients), and ART coverage, were compiled for 13 countries. An Ecological analysis using linear regression assessed which factors are associated with national variations in population female and male HIV prevalence, and with each other.

**Findings:**

National population HIV prevalence varies between 0 4–2 9% for men and 0 4–5.6% for women. ART coverage ranges from 6–23%. National variations in HIV prevalence are not shown to be associated with variations in HIV prevalence among FSWs or clients. Instead they are associated with variations in the percentage of younger and older males and females reporting multiple partners. HIV prevalence is weakly negatively associated with ART coverage, implying it is not increased survival that is the cause of variations in HIV prevalence. FSWs and younger female HIV prevalence are associated with client population sizes, especially older men. Younger female HIV prevalence is strongly associated with older male and female HIV prevalence.

**Interpretation:**

In West Africa, population HIV prevalence is not significantly higher in countries with high FSW HIV prevalence. Our analysis suggests, higher prevalence occurs where more men buy sex, and where a higher percentage of younger women, and older men and women have multiple partnerships. If a sexual network between clients and young females exists, clients may potentially bridge infection to younger females. HIV prevention should focus both on commercial sex and transmission between clients and younger females with multiple partners.

## Background: HIV in West Africa

In Sub-Saharan Africa, an estimated 23 5 million people are infected with HIV [1]. Whilst in West Africa HIV prevalence levels tend to be lower, Nigeria with a population of 178 million has an HIV prevalence of 3.1% and is the country with the second highest number of individuals infected globally. HIV prevalence in other countries in the region ranges from 0.5–4% [2].

There is substantial variation in population HIV prevalence within and between countries, with the almost universal practice of male circumcision in the region likely to be playing a central role in limiting the scale of the epidemic [3]. However, there are also substantial differences in the levels of infection between West African countries, with Nigeria, Cote d’Ivoire and Cameroon having estimated female population HIV prevalence levels ranging from 4–7%, compared to 0.5–2% more generally across the region [4].

Throughout West Africa, HIV remains largely concentrated in the most vulnerable populations [5], with transmission between female sex workers (FSWs) and their male clients thought to have a central influence on the scale of the epidemic [6,7]. Although population HIV prevalence remains relatively low [8], infection levels among brothel-based FSWs are far higher, ranging from 16% to 37% [9,10]. In such ‘concentrated’ HIV epidemic settings, prevention programming is focused on FSWs and their male clients [10,11], with the latter conceived as the main ‘bridging group’ through which HIV spreads to the general population [12,13].

However, such a simple programmatic focus may fail to respond to the complexity of sexual networks. Evidence suggests that a diverse group of women may exchange sex for money or resources, ranging from more formal commercial sex work that tends to be targeted by HIV programmes, through to informal transactional exchange, which may be more hidden [14–16]. Similarly, men who pay or provide resources for sex are a heterogeneous group [17,18]. As well as men who purchase sex from FSWs, men may provide money and/or resources to other long term and/or occasional sexual partners [19,20]. Multiple sexual partnerships are an important risk factor for HIV infection, if condoms are not used consistently [17,21]. Therefore, it is important to gain a clearer understanding of the relative epidemiological importance of commercial sex, versus the less visible dynamic of casual sex, which may or may not have a transactional element.

This paper presents the findings from an ecological analysis that explored whether the variations in population HIV prevalence observed across West Africa are associated with variations in the extent of formal commercial sex work, and/or the degree to which women and men of different ages report having multiple sexual partnerships in the past year. As the provision of anti-retroviral therapy (ARV) will also increase life expectancy (and so could potentially contribute to higher HIV prevalence levels), we similarly explore whether the variations in HIV prevalence may be associated with variations in national ARV coverage levels.

## Methods

A review of demographic health surveys (DHS) was used to compile recent epidemiological and behavioural data on patterns of sexual behaviour and levels of HIV infection for 2010–2014 amongst members of the general population. Most data were available from 13 West African countries. No DHS data were available for Ghana or Guinea Bissau and therefore these countries were excluded from analyses. A 2013 preliminary report for Gambia was available but contained only data on sexual behaviour and no HIV prevalence data. Data on brothel-based FSWs, were sourced from publications published after 2010 from regional sites within country settings. We included data on brothel-based FSWs only, since this group was easier to identify and categorise from the literature (S1 File).

For each country we extracted the most recent national estimates of the population prevalence of HIV among men and women nationally, and in urban and rural areas. Where available, we also extracted population data on the percentage of younger (15–24) and older (25–49) males and females reporting 2 or more (2+) partners in the past year (non-sexually active individuals were also included in the estimate), and the percentage of men reporting ‘paying for sex’ in the past year (assumed to be clients of FSWs). HIV prevalence among each sub-group was also compiled. In addition we collected data on condom use. Reported levels of condom use were relatively similar across countries for partnerships between key subgroups (S2 File) and FSW (also S1 File) and were therefore not considered within our analysis.

Estimates of the number of individuals receiving ART treatment (15–49) and the percentage of the population, 15 years or above who were HIV positive, were used to calculate the percentage of HIV infected people receiving ART treatment (S3 File). Population size estimates were sourced from the World Bank database [22], allowing for a 3% population growth rate (the average across West Africa).

Using this data a series of linear regression analyses were conducted in STATA 13 1 to identify which factors or independent variables were associated with the observed variations in HIV prevalence across West Africa. For this, a systematic approach was adopted where we explored whether factors hypothesised as being associated with population HIV prevalence (dependent variable), were significant. The independent variables included HIV prevalence in FSW and in clients, assuming that these factors may be independently associated with higher prevalence levels seen in the general population. We also assessed whether the percentage of younger and older males and females reporting 2+ partners in the past year, were significantly associated with higher HIV prevalence levels in the general population. The rationale for this being, that if there are more individuals with 2+ partners and they are at higher risk of acquiring HIV, then overall more infections will occur in the population, leading to higher levels of HIV prevalence.

In addition we explored whether HIV prevalence in younger and older females and males was associated with HIV prevalence in general population groups (of males and females). Here we wished to assess whether patterns of HIV observed in the younger age groups was correlated with general population prevalence, to understand patterns of infection and when infections are likely to occur. Finally, we analysed data on mean age at first sex to assess whether this variable was associated with the percentage of young males and females reporting 2+ partners.

Next we conducted regression analyses to explore patterns of association between different population subgroups. Our main objective for this was to explore whether the concept that HIV is predominantly transmitted along a pathway from FSWs, to clients to the general population is supported by empirical data. Firstly, we assessed whether HIV prevalence in FSWs and clients, treated as independent variables, was associated with HIV prevalence in subgroups of younger and older males and females with 2+ partners (dependent variables), i.e. does higher prevalence in these core groups directly impact on HIV prevalence in other high-risk subgroups. Next we assessed whether the percentage of clients in the population and younger and older males and females with 2+ partners were independently associated with HIV prevalence levels in FSWs, clients and younger and older males and females in the general population, i.e. are larger risk populations associated with higher prevalence levels in all groups.

Finally, we assessed the previous round of DHS data sets from 2003–2009 for countries across West Africa, to assess whether sexual behavioural patterns that pre-date the analysis have a delayed impact on HIV prevalence in the later surveys.

## Results

**Fig 1:**
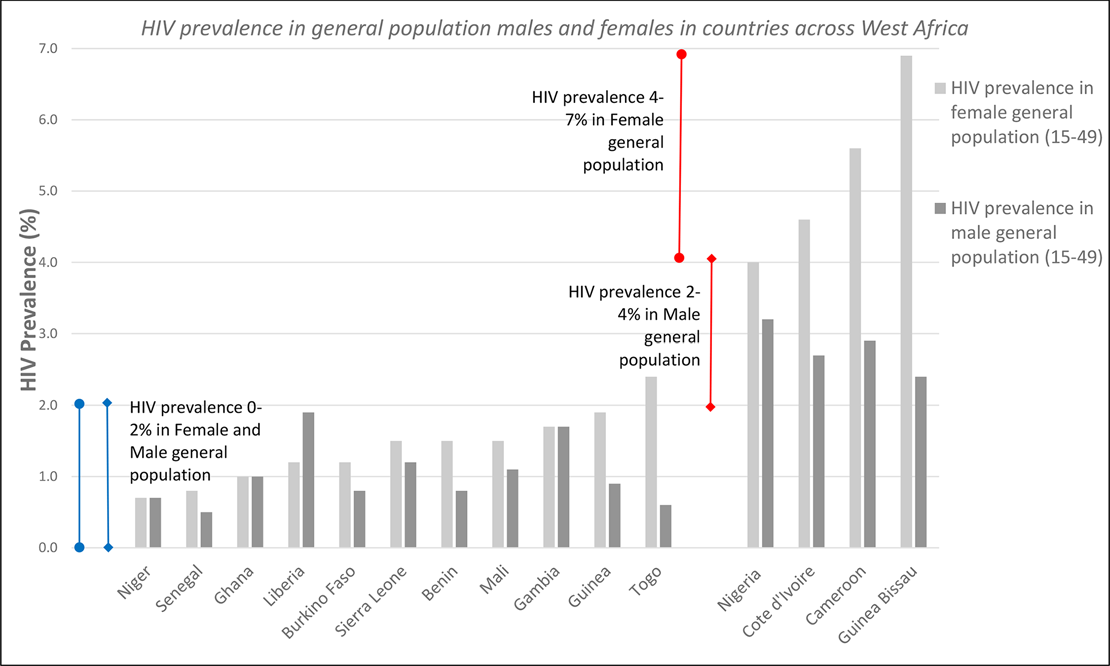
HIV prevalence data taken from DHS and UNGASS reports in West African settings from 2010–2014.

There is substantial variation in population HIV prevalence across the region for men (0.4–2.9%) and women (0 4–5.6%) respectively. Fig 1 shows how Cameroon, Cote d’Ivoire and Nigeria have the highest prevalence levels (4–5.6%) in general population females. For the remainder of countries, HIV prevalence tends to range from 0.4–2%. HIV prevalence is often higher in females than males and as expected, HIV prevalence is far higher among FSWs (15.9–36.7%).

**Fig 2:**
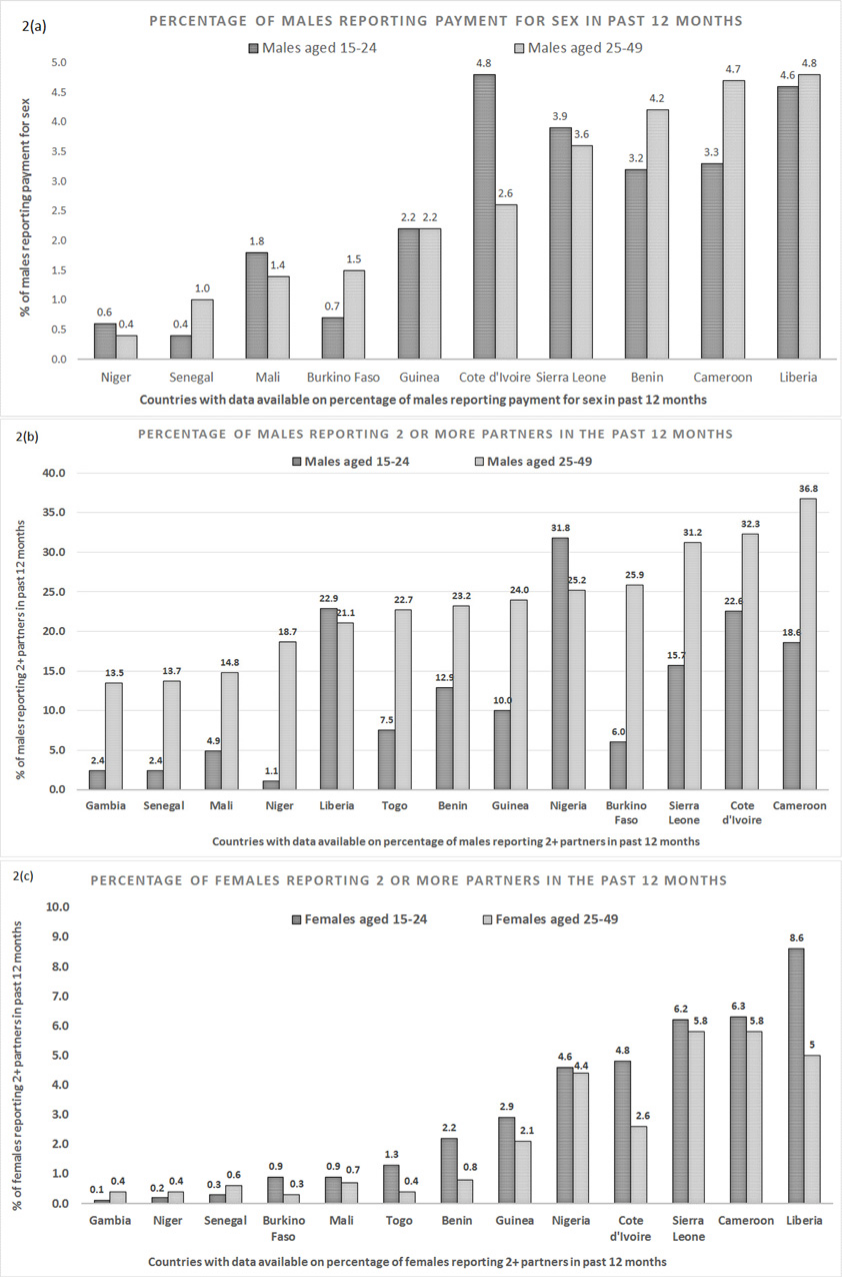
Fig 2(a): DHS Data from 10 West African countries, stratified by 15–24 and 25–49 year old males. Presented in ascending order of 25–49 year old males reporting payment for sex in the past 12 months, by country (2010–2014), data not available for Nigeria, Gambia and Togo. Fig 2(b): DHS data from 13 West African countries, stratified by age. Presented in ascending order of 25–49 year old males reporting 2 or more partners, by country. Fig 2(c): DHS data from 13 West African countries, stratified by age. Listed in ascending order of 15–24 year old females reporting 2+ partners in the past year, by country.

Fig 2(A) shows the percentage of males reporting payment for sex (clients) in the past year and compares the older males with the younger male group. We see that overall, levels tend to be low across countries (0.4% - 4.8%) with similar levels of younger and older males reporting payment being generally relative to one another in each country.

Fig 2(B) compares the percentage of younger and older males who report 2+ partners in the past year. With the exception of Liberia and Nigeria, a higher percentage of older males consistently report 2+ partners in the past 12 months (p = 0.002). For the male group the age of sexual debut ranges from 18.1–23.6 years with a negative correlation (p = 0.001) between age at first sex and percentage of young males reporting 2+ partners (S4 File). This may account for the lower percentage of younger males reporting 2+ partners in those countries.

A similar country pattern is seen amongst females (Fig 2(C)). However, for females the reverse pattern is seen, with a higher percentage of the younger group reporting 2+ partners compared to the older females. For younger females, age at first sex ranges from 15.9–19 years and is also negatively associated (Niger is removed as an outlier) with having 2+ partners (p = 0.03) (S4 File).

### Exploring the potential influence of variations in ART coverage and national HIV prevalence

From the available ART data there is an increase in coverage of ART from 2008–2012. In 2008 Burkina Faso had the highest coverage rate with approximately 12.5% of HIV positive individuals receiving treatment and Niger the lowest rate at 2.1%. The mean coverage across countries was 6.7%. By 2012 the mean coverage had risen to 13.5%, ranging from 7.2%-23.4%. The results from the regression analysis (S3 File) indicate a weak negative relationship between HIV prevalence and the percentage of individuals on ART. This suggests that levels of ART treatment within countries are unlikely to be responsible for differences in HIV prevalence levels.

### Associations between HIV prevalence among different-subgroups and male and female national, urban and rural HIV prevalence

Table 1 shows results from the linear regression analyses (p-values and R^2^) that assessed whether variations in national, urban and rural population HIV prevalence are associated with variations in the HIV prevalence or size of different subgroups. Significant associations (p<0 05) are shown in bold.

**Table 1:** Results from linear regression analysis. Associations between population subgroup HIV prevalence and relative population size, and levels of HIV infection in the general population (the number of countries included is shown in parenthesis).

	Variable (n = number of countries included in regression analysis)	Females						Males					
		Urban HIV prevalence		Rural HIV prevalence		National HIV prevalence		Urban HIV prevalence		Rural HIV prevalence		National HIV prevalence	
		R^2^	p	R^2^	p	R^2^	p	R^2^	p	R^2^	p	R^2^	p
**Brothel based FSWs**	HIV prevalence (10)	0.39	0.08	0.31	0.11	0.44	0.06	0.31	0.11	0.25	0.17	0.34	0.10
**Brothel based FSWs**	% with risk behaviour	na	na	na	na	na	na	na	na	na	na	na	na
**Males reporting payment for sex in past year**	HIV prevalence 15–49 (10)	0.001	0.95	0.06	0.53	0.001	0.95	0.001	0.94	0.05	0.57	0.003	0.88
**Males reporting payment for sex in past year**	% with risk behaviour aged 15–49	0.31	0.1	0.27	0.31	0.37	0.06	**0.64**	**0.005**	0.23	0.16	**0.54**	**0.02**
**Males reporting payment for sex in past year**	% with risk behaviour aged 15–24	-0.34	0.08	0.22	0.17	0.38	0.05	**0.79**	**0.0005**	0.28	0.12	**0.58**	**0.01**
**Males reporting payment for sex in past year**	% with risk behaviour aged 25–49	0.24	0.16	0.26	0.13	0.31	0.09	**0.46**	**0.03**	0.12	0.23	**0.43**	**0.04**
**15–24 year old females**	HIV prevalence (12)	**0.44**	**0.01**	**0.76**	**0.0002**	**0.68**	**0.0001**	**0.85**	**0.0001**	**0.85**	**0.0001**	**0.87**	**0.0001**
**25–49 year old females**	HIV prevalence (12)	**0.93**	**0.0001**	**0.82**	**0.0001**	**0.97**	**0.0001**	**0.61**	**0.003**	**0.65**	**0.001**	**0.78**	**0.0001**
**15–24 year old males**	HIV prevalence (12)	0.01	0.79	0.25	0.10	0.08	0.36	0.30	0.07	**0.40**	**0.03**	0.30	0.07
**25–49 year old males**	HIV prevalence (12)	**0.79**	**0.0001**	**0.87**	**0.0001**	**0.95**	**0.0001**	**0.84**	**0.0001**	**0.83**	**0.0001**	**0.96**	**0.0001**
**15–24 year old females 2+ partners in past 12 months**	% with risk behaviour (12)	0.21	0.12	0.22	0.13	0.30	0.06	**0.49**	**0.01**	0.18	0.16	**0.44**	**0.019**
**25–49 year old females 2+ partners in past 12 months**	% with risk behaviour (10)	**0.64**	**0.005**	**0.88**	**0.0001**	**0.85**	**0.0002**	**0.76**	**0.001**	**0.78**	**0.0008**	**0.86**	**0.0001**
**15–24 year old males 2+ partners in past 12 months**	% with risk behaviour (12)	0.23	0.11	**0.46**	**0.02**	**0.43**	**0.02**	**0.80**	**0.0001**	**0.57**	**0.0047**	**0.74**	**0.003**
**25–49 year old males 2+ partners in past 12 months**	% with risk behaviour (10)	0.31	0.09	**0.70**	**0.003**	**0.56**	**0.01**	**0.83**	**0.0003**	**0.83**	**0.0003**	**0.82**	**0.0003**

Data in bold for p<0.05. * Subgroup population size data for Liberia and Sierra Leone were both significant outliers in the regression analysis for females 15–24 and 25–49 with 2 or more partners in the past 12 months. We therefore performed a second round of analyses for the 2+ partner groups where these were excluded, to compare results across both the male and female subgroups with 2+ partners.

The findings show that brothel-based FSW HIV prevalence is not strongly associated with the HIV prevalence in any other population group. This is also true for the HIV prevalence in male clients. However, there is a significant association between the *size* of the client groups (in particular the younger group) and HIV prevalence in males at the urban and national levels.

As expected HIV prevalence among younger and older females and older males is significantly correlated with urban, rural and national HIV prevalence. However, there was only a very weak correlation between HIV prevalence among younger males and female HIV prevalence at a rural, urban or national level, and with only weak (ranging from p = 0.03 for rural males to p = 0.07 for urban males and at a national level) associations with male HIV prevalence levels.

Next, we considered the potential influence on variations in national, urban and rural HIV prevalence of the size of younger and older males and females reporting 2+ partners or buying sex (for men) in the past 12 months. For females, when all countries were included in the regression, the percentage of both younger and older females having 2+ partners was only significantly associated with urban male HIV prevalence. However, when the two outliers, Liberia and Sierra Leone, were removed from the analyses, the percentage of younger and older females reporting 2+ partners was significantly positively (p<0.005) associated with variations in national, urban, and rural HIV prevalence among males and females. For males, regression analysis for those including and excluding Liberia and Sierra Leone, gave similar results, showing that the percentage of both younger and older males reporting 2+ partners were significantly associated with HIV prevalence among all population groups.

Given the strong associations between population HIV prevalence and the size of the subgroups reporting 2+ partners in the past 12 months, we explored which factors were associated with variations in HIV prevalence among the different sub-populations considered. Tables 2 and 3 summarise these results. Table 2 shows the relationships between HIV prevalence in different groups. Firstly, there are no significant associations between the HIV prevalence in male clients (15–49) nor younger males and other population groups. FSW HIV prevalence is correlated with HIV prevalence among both older males and females, but not with any other groups. Finally, there is strong evidence for an association between HIV prevalence in young females (despite a lack of association in their young male counter-parts) and both groups of older males and females. As expected HIV prevalence in older males and females is correlated.

**Table 2:** Results from regression analysis showing level of association (p-values) from the linear regression analysis for associations between levels of HIV prevalence in different subgroups.

Table 2 HIV Prevalence						
(n = number of countries included in regression analysis)	Brothel Based Female Sex Workers	Men Who Report Payment for Sex (15–49)	15–24 year old females	15–24 year old males	25–49 year old females	25–49 year old males
Brothel Based Female Sex Workers (10)	-	-	-	-	-	-
Men Who Report Payment for Sex (15–49) (10)	0.16	-	-	-	-	-
15–24 year old females (12)	0.10	0.17	-	-	-	-
15–24 year old males (12)	0.86	0.22	0.01*	-	-	-
25–49 year old females (12)	**0.002**	0.29	**0.01**	0.62	-	-
25–49 year old males (12)	**0.01**	0.32	**0.001**	0.16	**0.001**	

**Table 3:** Results from regression analysis showing the level of association (p-values) from the linear regression analysis between the size of different subgroups in the population and HIV prevalence amongst the subgroups. Significant relationships (p<0.05) are in bold text.

**Table 3 HIV Prevalence**						
(n = number of countries included in regression analysis)	Brothel Based Female Sex Workers	Men Who Report Payment for Sex (15–49)	15–24 year old females	15–24 year old males	25–49 year old females	25–49 year old males
Men Who Report Payment for Sex (15–49) (10)	0.2	0.06	0.17	0.22	0.09	0.07
Men Who Report Payment for Sex (15–24) (10)	**0.05**	-	**0.01**	0.06	0.18	0.08
Men Who Report Payment for Sex (25–49) (10)	**0.009**	-	**0.005**	**0.01**	0.12	0.08
15–24 year old females 2+ partners (10)	**0.004**	0.29	**0.003**	0.19	**0.001**	**<0.001**
15–24 year old males 2+ partners (10)	**0.05**	0.28	**0.001**	**0.01**	**0.05**	**0.003**
25–49 year old females 2+ partners (10)	**0.04**	0.93	**0.001**	0.11	**0.008**	**0.002**
25–49 year old males 2+ partners (10)	**0.001**	0.06	**0.04**	0.48	**0.003**	**0.008**

Table 3 explores the association between HIV prevalence in different sub-groups and their behaviour. The HIV prevalence in young females and in FSWs are associated with the size of both the younger and older group of male clients, and with the size of both younger and older groups of males and females who report 2+ partners in the past year. Yet, we see little evidence for an association with HIV prevalence in male clients or in the younger males reporting 2+ partners and the size of other population subgroups.

Exploring significant relationships between the sizes of population subgroups, we observe a strong association between the size of the older group of male clients and HIV prevalence among (i) FSWs, (ii) younger males and younger females. For the young male client group, there is only an association with HIV prevalence in FSWs and younger females reporting 2+partners.

### Assessing evidence for a time lag in HIV prevalence as a result of earlier high-risk sexual behaviour

Because of the apparent time lag in high-risk behaviours impacting on HIV prevalence at a later date, we also reviewed the data from earlier DHS surveys from 2003 to 2009. We compared this with the data used in the analysis from 2010 to 2014. Due to revisions in the questionnaire, several of the earlier questionnaires did not include questions on 2+ partnerships. Despite this, two key observations are evident from the six country studies that could be compared. Firstly, HIV prevalence across this period tended to remain fairly constant in countries with lower prevalence levels, with the exception of Liberia where prevalence fell from 1.8% to 1.2% in general population females. For the countries where HIV prevalence is higher, Cameroon and Cote d’Ivoire, prevalence also declined from 6.8% to 5.6% and 6.4% to 4.6%, respectively. For the five countries with data available on 2+ partners in both rounds, either a decline in this behaviour or no change was also observed. In Liberia, the percentage of young females reporting 2+ partners declined from 9.5% to 8.6% and in Cote d’Ivoire from 6.2% to 4.8%. For the remaining countries (Benin, Guinea and Sierra Leonne) there was no reported change in behaviour. Here, we see that declines in HIV prevalence seem to mirror changes in high-risk behaviours, amongst the female 2+ group. However, there is no clear indicator of a delay in behaviour leading to a reduction in HIV prevalence, since both variables appear to decline concurrently.

## Discussion

This study has sought to identify which ecological variables are associated with the 6–10 fold variation in population HIV prevalence across West Africa–a region where male circumcision is widespread.

Our analysis has several main limitations. The first is the ecological nature of the analysis, as we compare patterns of association using aggregated national data coming from multiple sources. In practise the multiple data sources are unlikely to be a major limitation, as most of the behavioural and HIV data used came from national DHS surveys, with some comparability internally and between countries. For each country we used the most recent data available, and the 4 year timespan will only have influenced our findings if HIV prevalence levels and/or patterns of population sexual behaviour changed substantially over this time. This is difficult to assess in the absence of data. However, given the ecological nature of this analysis, the findings should be seen as hypothesis generating, rather than providing strong evidence of causality.

Secondly, reporting bias is likely to have influenced our estimates of the size of different sub-populations. Questions on sexual behaviour—including on men’s purchasing of sex or the numbers of sexual partners–are highly sensitive, and prone to under-reporting, especially among women [23]. For this reason, our values will be under-estimates [21]. If there is no substantial differences in the degree of under-reporting between countries, the findings may nevertheless reflect true variations between countries. If there is also varying degrees of under-reporting between countries, we cannot rule out this hidden confounder. However, such misclassification is likely to weaken associations found in regression analyses, and so if anything, will lead to null findings. Given this, we expect that significant associations are meaningful, but caution against over-interpreting the lack of associations seen.

More broadly, the forms of analysis that we could conduct were limited by available data. There is multiple heterogeneity in patterns of sexual behaviour between countries. We were limited to using variables extracted from national DHS and other data sets. In particular, some variables (such as the size of the female sex worker population) was only available for a limited number of countries considered; HIV prevalence data on males reporting payment for sex was not disaggregated by age; we were not able to assess the degree to which men’s reporting of purchasing sex related primarily to engagement in commercial sex, or other forms of sexual exchange; the degree to which young girls have sex with men with a history of multiple partners, consider whether sex worker and/or client migration may be important; or explore the influence of other factors, such as male sex work and injecting drug use.

Despite these limitations, we found several strong associations. Variations in population HIV prevalence across West Africa are strongly associated with variations in the extent that males and females in the general population report 2+ partners in a 12 month period. Stratification (into 15–24 and 25–49 year olds) by age and further analysis of these subgroups reporting 2+ partners in the past year reveals that HIV prevalence in younger females and female sex workers is strongly associated with the size of both male and female populations reporting 2+ partners in the past year. However, data on brothel-based FSWs within countries was collected mostly within specific locations or districts, often using different sampling criteria and parameters, so we must caution over-interpretation for this set of results.

Additionally we see that the percentage of males in the population reporting payment for sex (in the past 12 months), especially amongst the older males is associated with HIV prevalence in younger males and females as well as FSWs.

From the earlier rounds of DHS data we did not observe any clear evidence for a time lag in sexual behaviour, impacting on HIV prevalence. A reason for this is that it is likely that behaviour changes involving multiple partners, increases in condom use and other preventative methods may pre-date these surveys and as such, here we observe the impact of these. Historical data suggests the HIV epidemic in West Africa peaked around 1999–2000 in most countries [24], although in the absence of reliable early data it is difficult to verify this behaviour change.

**Fig 3:**
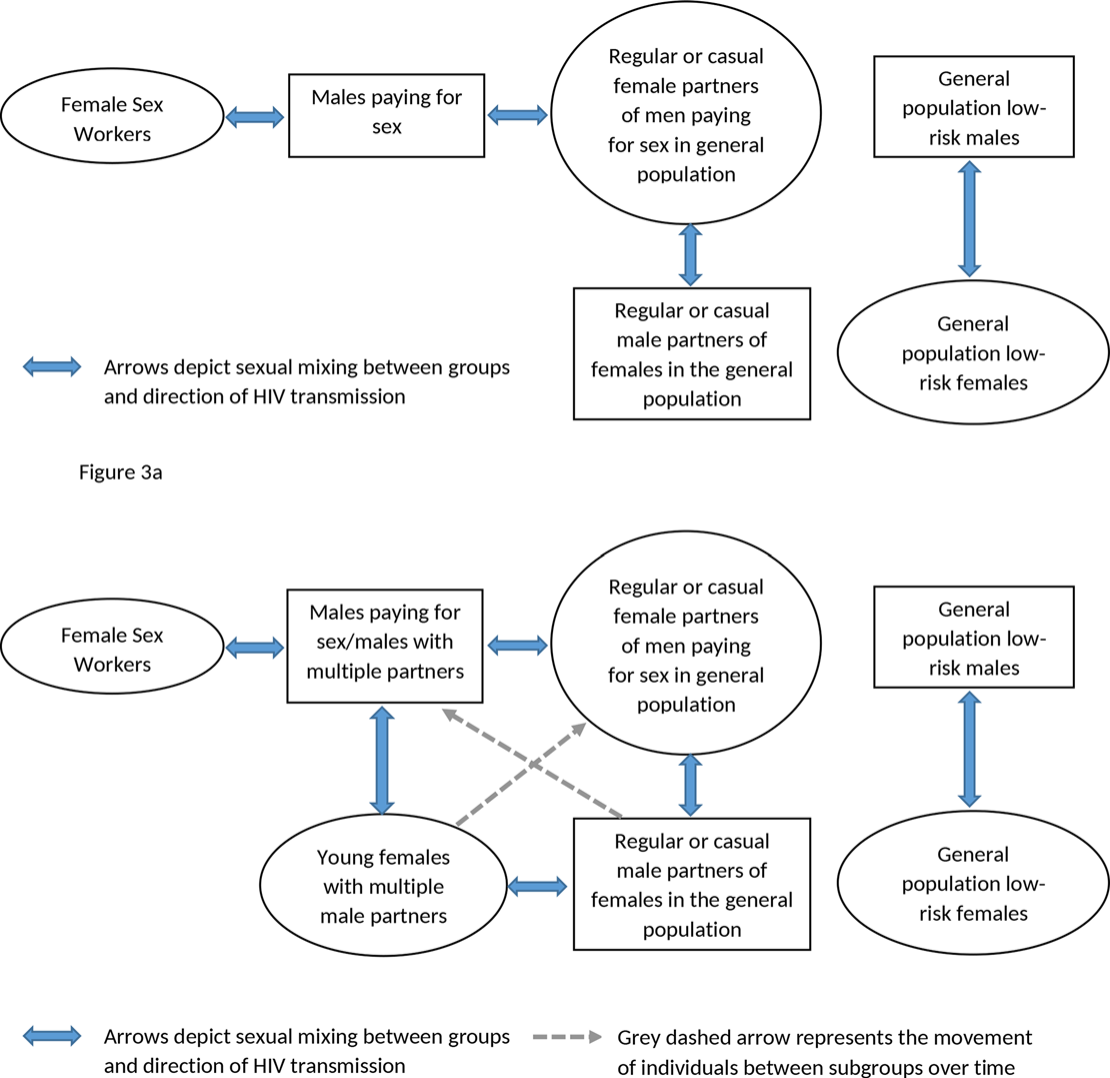
Fig 3(a): Conceptual pathway of heterosexual HIV transmission from female sex workers to the general population in West Africa and Fig 3(b) Revised conceptual framework of HIV spread through sexual networks of individuals within the population.

The findings have implications for the way in which epidemiologists understand the determinants of HIV epidemics. Fig 3(A) visually depicts the dominant epidemiological theory of HIV transmission in heterosexual concentrated epidemics [25,26]. Epidemiological models and HIV programmes are often constructed on the assumption that HIV is transmitted from FSWs to men who purchase sex (clients), with the multiple encounters of a large group of men with a small ‘core’ of sex workers providing a context in which rapid HIV transmission may occur (the core group theory) [27]. In turn, HIV then passes from these male clients to their steady or casual female partners in the general population, with clients being an important ‘bridging population’ for HIV transmission [26].

Fig 3(B) shows a revised theory, emerging from this analysis. This is similar to 3(a), but also highlights that sexual activity between higher risk men (such as clients) and young girls may provide an important additional bridge of infection into the general population. In this revised theory, the proportion of adolescent females with multiple partnerships who have sex with high-risk men are at higher risk of HIV infection than other young females. Onward transmission may subsequently occur if girls also partner with lower risk men or re-infect higher-risk partners, either casually, or as they enter more stable partnerships. In this way, the higher HIV levels among these young women then leads to higher infection rates in all men. Over time also, adolescent females age into the general population, and so influence population HIV prevalence, especially if this group is large and survival is high.

In this revised theory, the size of the HIV epidemic is dependent on the extent that ‘men pay for sex and/or have multiple partners’, the extent that young girls have multiple partnerships, and the levels of ‘connectivity’ of these groups. Populations with large proportions of young females having multiple partners and large proportions of males reporting payment for sex or multiple partners would tend to have higher levels of HIV prevalence. It may also explain why there is a greater disparity between male and female HIV prevalence in countries with higher HIV prevalence levels, because of the higher incidence rate in young females. Conversely, in countries where HIV epidemics remain at lower levels and are more concentrated, it may be that the proportion of young females with multiple partners is not large enough or not ‘connected’ enough to higher risk men, to have a large contribution to the HIV epidemic. This may be the case for Liberia and Sierra Leone. For example, Liberia the only country in the analysis where male HIV population prevalence is greater than in females, suggesting possibly a different dynamic in the transmission pathway for HIV. We are currently conducting epidemiological modelling to explore these issue further.

## Conclusion

The observed variation in population HIV prevalence across West Africa is strongly associated with national variations in the extent that men buy sex and individuals have multiple partners. The results from the regression analysis appear to show a linear trend between the percentage of individuals who report 2+ partners and HIV in the general male and female population. Interestingly, however, it is only the HIV prevalence in younger females and among brothel-based female sex workers that is associated with both multiple partnerships (2+ partners) in the population and males reporting payment for sex. This may highlight an important link between these population groups. Females with 2+ partners may form a critical bridge between high-risk men and the general population, that helps sustain larger HIV epidemics. In these countries prevention should focus both on commercial sex and adolescent girls with multiple partners.

The findings illustrate the importance of continually monitoring the distribution of HIV infection, and patterns of sexual behaviour, and the need to use this information to inform the efficient use of HIV prevention resources.

The results also support dominant epidemiological thinking about the important role of commercial sex to the HIV epidemic in West Africa, as highlighted in a recent modelling study [28]. There is substantial programmatic experience with the provision of HIV prevention to sex workers and their clients, and multiple examples of where such programming has had marked impacts on the HIV epidemic [29,30]. It is important that such programmes are sustained and expanded, and that opportunities to achieve greater impacts, for example, through the additional provision of ART based prevention technologies, are explored.

Our findings also highlight that sexual activity between high-risk men and young girls is an important additional route through which HIV infection may spread to the general population. This poses major challenges for HIV programmes, as girls with multiple partners who may be involved in transactional exchanges (both monetary and material) but who do not identify themselves as sex workers, can be difficult to identify and reach. A number of potentially promising intervention models for adolescents are starting to emerge in other African regions. This includes the Zomba trial in Malawi, that showed an impact on HIV by providing cash transfers to keep girls in school [31] interventions that provide information about the risks of ‘risky male’ partners [32], and interventions which aim to socially and economically empower women [33]. There is an urgent need for interventions to address HIV risk among adolescent girls and their sexual partners in the West African context, especially, where multiple partnerships are more commonly reported. With recent studies showing a lack of association between age disparate relationships between older males and younger females [34], it is important to understand the mechanisms through which young females become infected in such high numbers.

## Supporting Information

S1 File (a) Condom use in population subgroups from DHS surveys 2010–2014. (b) Age at sexual debut and association with percentage of males and females reporting 2+ partnerships in population. (c) Literature review Female Sex Worker: Relative population size, condom use, HIV prevalence. Quantitative and qualitative research studies were searched in Pubmed, Adolec, and Popline, using the following search terms: ‘sex work’, ‘sex worker’, ‘prostitute’, ‘prostitution’, ‘transactional sex’. In addition, when no information was available using these search terms, the term ‘HIV’ was used. The search was limited to the 2010–2013 time period. Abstracts were further examined to determine eligibility for inclusion. In addition, grey literature and reports, such DHS, IBSS, UNGASS, UNAIDS, UNICEF, USAID, World Bank were studied as far as they were accessible.
(PDF)

S2 File Figures on Anti-Retroviral treatment for countries across West Africa. (PDF)

S3 File Results from the regression analysis showing significant relationships (p<0.05, highlighted in grey) for variables associated with variations in HIV prevalence among different sub-populations. (PDF)

S4 File Age at sexual debut and association with percentage of males and females reporting 2+ partnerships in population. (PDF)
