# Supplementary material for: Factors Associated with Variations in Population HIV Prevalence across West Africa: Findings from an Ecological Analysis
Source: PLoS One. 2015 Dec 23;10(12):e0142601. doi: 10.1371/journal.pone.0142601 (PMC4689529; doi:10.1371/journal.pone.0142601)
Supplement: S1 File — Quantitative and qualitative research studies were searched in Pubmed, Adolec, and Popline, using the following search terms: ‘sex work’, ‘sex worker’, ‘prostitute’, ‘prostitution’, ‘transactional sex’. In addition, when no information was available using these search terms, the term ‘HIV’ was used. The search was limited to the 2010–2013 time period. Abstracts were further examined to determine eligibility for inclusion. In addition, grey literature and reports, such DHS, IBSS, UNGASS, UNAIDS, UNICEF, USAID, World Bank were studied as far as they were accessible. (PDF) [file pone.0142601.s001.pdf]

## S1 File– Literature review Female Sex Worker: Relative population size, condom use, HIV prevalence.

Quantitative and qualitative research studies were searched in Pubmed, Adolec, and Popline, using the following search terms: ‘sex work’, ‘sex worker’, ‘prostitute’, ‘prostitution’, ‘transactional sex’. In addition, when no information was available using these search terms, the term ‘HIV’ was used. The search was limited to the 2010-2013 time period. Abstracts were further examined to determine eligibility for inclusion. In addition, grey literature and reports, such DHS, IBSS, UNGASS, UNAIDS, UNICEF, USAID, World Bank were studied as far as they were accessible.

**Benin:** population 8 525 574 (UNGASS 2012/2010)

| **Parameter variable** | **Estimate** | **Source** | **Study detail** | **Comment** |
|---|---|---|---|---|
| Consistent condom use FWSs with clients | 91·7% last seven days<br>85·1% last day of work (91,7% among official FSWs, 80,2% among clandestine) | [1] | 1016 FSWs | |
| | 94·6% always during last 7 days | [2] | 773 FSWs (15-29) were interviewed 429 official and 344 clandestine | |
| HIV prevalence FSWs | 20·4%· 25,2% among official FSWs and 17,7% among clan destines | [1] | 1016 FSWs | |

**Burkina Faso: Population**: 14 017 267 (2006)

| **Parameter variable** | **Estimate** | **Source** | **Study detail** | **Comment** |
|---|---|---|---|---|
| Consistent condom use FSWs clients | 98·2% last client<br>94·8% last day<br>92·8% always | [3] | Sample=1016 FSWS<br><br>62% seaters 21% street FSWs, 18% clandestine. | From: l’Enquête bio comportementale auprès des travailleuses de sexe et leurs clients<br><br>94% of clandestine FSWs were from particular province Poni |

| | | | | |
|---|---|---|---|---|
| | 89·7% last new client<br>78·3% last regular client | 4 | 115 professional SWs of the ANRS 1222 Yerelon Cohort at Enrolment | At the base-line |
| HIV prevalence FSWs | 25·7%<br>35·9% among professional SWs<br>23·5% among part-time SWs | 4 | 658 FSWs (full-time and part-time) of the ANRS 1222 Yerelon Cohort at Enrolment | Mean age 25 |

**Cameroon: Population:** 22.25 million (2013)

| **Parameter variable** | **Estimate** | **Source** | **Study detail** | **Comment** |
|---|---|---|---|---|
| Consistent condom use FSW | 72·7% last paid sex | 5 | | |
| HIV prevalence FSWs | 36·7% 2009 | 5 | | |
| | 36%, the highest in Adamoua region-49.5%; the lowest in Sud region-23.9%<br>The highest prevalence was among 20-29 years old FSWs | 6 | | |

**Cote d'Ivore:** Population 19 737 800 (2010)

| **Parameter variable** | **Estimate** | **Source** | **Study detail** | **Comment** |
|---|---|---|---|---|
| Consistent condom use FSWs clients | 92·8% last client | 7 | | Enquête CAP PS 2011 dans 12 régions |
| HIV prevalence FSWs | 26·6% | 8 | Cross-sectional survey among 1110 FSWs who attended clinics in 4 regions in 2007 and in 2009 | Same study cited in UNGASS report but with prevalence 28·6% |

**Gambia:** population**:** 1 776 000 (2011) World Bank

| **Parameter variable** | **Estimate** | **Source** | **Study detail** | **Comment** |
|---|---|---|---|---|
| Consistent condom use FSWs | 96·7% last sex | 9 | | |

**Ghana:** population 24 223 430 (census 2010)

| **Parameter variable** | **Estimate** | **Source** | **Study detail** | **Comment** |
|---|---|---|---|---|
| Percentage of FSWs | 51,934 (47,786 – 58,920)<br>0·7% of females aged 15-64 | 10 | National level<br>Mapping | From: IBBSS, 2011<br><br>90% of those were roamers |
| Consistent condom use FSWs clients | 92% last client<br><br>90·3% among roamers and 95·9% among seaters | 10 | | From: IBBSS, 2011 |
| | 79·2% always<br><br>74·6% among roamers and 89·9% among seaters | 10 | | From: IBBSS, 2011 |
| | 90·2% with clients in last 3 months | 11 | 559 FSWs<br>(75% roamers, 25% seaters)<br>mean age 27 in Tema-Paga transport corridor | |
| HIV prevalence FSWs | 25·1% | 10 | | Declined from 34% in 2006 |

**Guinee**: population 10 200 000 (UNGASS 2012)

| **Parameter variable** | **Estimate** | **Source** | **Study detail** | **Comment** |
|---|---|---|---|---|
| Consistent condom use FSWs clients | 77% last client | 12 | National survey | |

| | | | | |
|---|---|---|---|---|
| | 84·1% during last 12 months | [13] | National survey among 1009 FSWs | |
| HIV prevalence FSWs | 34·4% | [12] | | From: Enquête nationales auprès des population à Risqué (ESCOMB 2007) |
| | 32·7% | [12] | In Corridor Boke among 101 tested FSWs | From: Enquête regionale faite sur un échantillon réduit dans trois regions<br>It was 20·7% in the same region in 2007 (among 598 FSWs) |

**Liberia:** population 3 500 000 (Census, 2008)

| Parameter variable | Estimate | Source | Study detail | Comment |
|---|---|---|---|---|
| Percentage of FSWs | Estimated size 1822 (about 0·2% among 15-49 age) | [14] | Data collected in 2011 | This was a size estimate study in 2011 among MARPs |

**Mali:** population 14 517 176 (UNGASS, 2012, data 2009)

| Parameter variable | Estimate | Source | Study detail | Comment |
|---|---|---|---|---|
| Consistent condom used FSWs with clients | 98·1% last sex | [15] | | From: ISBS, 2009 |
| HIV prevalence FSWs | 24·2% | [15] | | From: ISBS, 2009 |

**Niger:** population 14 517 176 (UNGASS, 2012, data 2009)

| Parameter variable | Estimate | Source | Study detail | Comment |
|---|---|---|---|---|
| Consistent condom used FSWs with clients | 94·4% last sex | [16] | | From: Enquête SSG 2011 |

| | | | | |
|---|---|---|---|---|
| HIV prevalence FSWs | 35·6% | 16 | N=900 | From: Surveillance sentinelle 2009<br>In different regions from 16,7% to 60,6% |

## Nigeria

Population: 162,265,000 (UNGASS, 2012)
Note: most data comes from last IBBSS conducted in nine Nigerian states in 2010: Lagos, Kano, Kaduna, Benue, Nasarawa, Edo, Anambra, Cross River, FCT. Study recruited 2, 265 BB-FSWs and 2,194 NBB-FSWs

| **Parameter variable** | **Estimate** | **Source** | **Study detail** | **Comment** |
|---|---|---|---|---|
| Consistent condom use BB-FSWs with client | 95·1% (last sex<br>90·6% (last 30 days) | 17 | | |
| HIV prevalence BB-FSWs | 27·4% | 17 | | HIV was higher in females with no formal education and in females who reported spending 5+ years in sex work |
| | 29·6% | 18 | N=124 FSWs from Kano region, average age 26·4 | |
| HIV prevalence NBB-FSWs | 21·1% | 17 | | HIV was higher in females with primary education and in females who reported spending 5+ years in sex work |
| HIV prevalence among transport workers | 2·4% | 17 | | |
| HIV prevalence among armed forces | 2·5% | 17 | | |
| HIV prevalence among police | 2·6% | 17 | | |

**Senegal:** population**:** 12 855 153 (2011)

| **Parameter variable** | **Estimate** | **Source** | **Study detail** | **Comment** |
|---|---|---|---|---|
| Consistent condom use FSWs clients | 93·7% last client<br><br>94·6% last old client among professional SWs<br>90·7% among clandestine | 19 | N=703 | From: ENCS 2010 |

| | | | | |
|---|---|---|---|---|
| | 98·7% new client among professional SWs<br>95·7% among clandestine | | | |
| HIV prevalence FSWs | 18·5%<br>21·6% among professional SWs<br>9·9% among clandestine | 19 | N=703 | It was 19·8% in 2006<br>23·8% among official sex workers<br>12·1% among clandestine |

**Sierra Leone:** population 5 600 000

| Parameter variable | Estimate | Source | Study detail | Comment |
|---|---|---|---|---|
| Percentage of FSWs | 3·5 % (82,779) | 20 | | |

**Togo:** population 6 200 000 (UNGASS, 2012/data 2010) 25% women 15-49 (795,515)

| Parameter variable | Estimate | Source | Study detail | Comment |
|---|---|---|---|---|
| Percentage of FSWs | 5397 (0,7% of 15-49 females) | 21 | Data was collected in 2005 in all Togo regions at SW sites and included both BB and NBB-FSWs | |
| Consistent condom use BB-FSWs and NBBs | 95·3% ever<br>88% consistently during last day of sex work<br>79·1% always during the last week | 22 | 1106 FSWs | |
| HIV prevalence BB-FSWs and NBBs | 13·1% (21·8 among official FSWs and 10% among clandestines)<br>28% among 18-24 year olds | 22 | Among 294 official sex workers and 812 clandestine sex workers | |

## References


1. Programme National de Lutte contre le SIDA et les IST DNdlSP, Ministère de la Santé. Enquete de surveillance de deuxieme generation des IST/VIH/SIDA au Benin (ESDG-2012): TOME 1 : Travailleuses du sexe et Serveuses de bars/restaurants. 2012.
2. Division PR. Bénin (2011) : Evaluation de l'utilisation du condom avec des partenaires payants chez les Travailleuses de Sexe de 15-29 ans des zones d'intervention du projet IMPACT, 3ème PASSAGE. PSI Social Marketing Research Series, 2011.
3. Publique'96 BdAeS. enquête de surveillance comportementale auprès des groupes a haut risque : travailleuses du sexe (ts) et leurs clients au burkina faso. burkina faso: cnls, 2011.
4. Konate I, Traore L, Ouedraogo A, Sanon A, Diallo R, Ouedraogo JL, et al. Linking HIV prevention and care for community interventions among high-risk women in Burkina Faso--the ARNS 1222 "Yerelon" cohort. Journal of acquired immune deficiency syndromes. 2011 Jul 1;57 Suppl 1:S50-4
5. UNGASS. Cameroun Rapport de progres n°3 comite national de lutte contre le sida national aids control committee mise en œuvre de la declaration d'engagement sur le vih/sida. republique du cameroun: comite national de lutte contre le sida 2012.
6. UNAIDS, UNFPA-Cameroun. Enguete seroepidemiologique et comportementale sur le VIH et la syphilis chez les travailleurs du sexe au Cameroun. 2010.
7. SIDA CNdLCl. Rapport National GARP Côte d'Ivoire. 2012
8. Vuylsteke B, Semde G, Sika L, Crucitti T, Ettiegne Traore V, Buve A, et al. HIV and STI prevalence among female sex workers in Cote d'Ivoire: why targeted prevention programs should be continued and strengthened. PloS one. 2012;7(3):e32627
9. President NASOoT. Country progress report: the Gambia. 2012.
10. UNAIDS. Ghana country AIDS progress report: reporting period 2010-2011. 2012.
11. International Organization for Migration (IOM) G. Behavioural Study of Female Sex Workers along Ghana's Tema-Paga Transport Corridor. International Organization for Migration, Ghana: 2012.

12. SIDA CNdLcl. Rapport UNGASS 2012-Guinée. UNAIDS, 2012.

13. Metrics PR. Guinée (2010): Mesure de la couverture et de la qualité du Préservatif Prudence Plus dans les sites de prostitution en Guinée. PSI Social Marketing Research Series, 2010.

14. UNAIDS. United Nations General Assembly Special Session on HIV and AIDS: Country Progress Report, 2012, Republic of Liberia. 2012.

15. SIDA Hcndlcl. Rapport national UNGASS 2012: Période Janvier 2010 - Décembre 2011. UNAIDS, 2012.

16. SIDA CNdlcl. Rapport d'activite sur la riposte au SIDA au Niger: période considérée Janvier 2010 - Décembre 2011. UNAIDS, 2012.

17. (FMOH) FMoH. HIV Integrated Biological and Behavioural Surveillance Survey (IBBSS) 2010. 2010.

18. Lawan UM, Abubakar S, Ahmed A. Risk perceptions, prevention and treatment seeking for sexually transmitted infections and HIV/AIDS among female sex workers in Kano, Nigeria. African journal of reproductive health. 2012 Mar;16(1):61-7

19. SIDA Cndlcl. Rapport de situation sur la riposte nationale a l'epidemie de situation sur de VIH/SIDA Senegal: 2010-2011. 2012.

20. Kenya P.R. RA, Kobba J., Conteh N., Thompson D., Kamara V., Lwamba C. HIV Modes of Transmission and Prevention Response Analysis. 2010.

21. Sobela F, Pepin J, Gbeleou S, Banla AK, Pitche VP, Adom W, et al. A tale of two countries: HIV among core groups in Togo. Journal of acquired immune deficiency syndromes. 2009 Jun 1;51(2):216-23

22. IST PNdLclSel. Enquête comportementale et de séroprévalence du VIH chez les professionnels de sexe et leurs clients au Togo en 2011. 2011.
