# Supplementary material for: Factors Associated with Variations in Population HIV Prevalence across West Africa: Findings from an Ecological Analysis
Source: PLoS One. 2015 Dec 23;10(12):e0142601. doi: 10.1371/journal.pone.0142601 (PMC4689529; doi:10.1371/journal.pone.0142601)
Supplement: S2 File — (PDF) [file pone.0142601.s002.pdf]

S2 File – Condom use in population subgroups from DHS surveys 2010-2014

	Females 2+ (%)	Males 2+ (%)	Males Payment (%)
Benin	30.1	19.9	59.9
Burkino Faso	62.3	22.2	32.4
Cameroon	37.3	39.6	53.2
Cote d'Ivoire	29.7	32.9	61.8
Gambia		18.8	
Guinea	32.1	25.4	
Liberia	19.6	23.6	55.9
Mali	9.9*	10.1*	61.2
Niger		22.9[#]	56.8
Nigeria			
Senegal		20.7	
Sierra Leone			
Togo	55.4	23.9	

*Data was heavily biased towards condom reporting from rural population

[#]Data representative of urban populations because actual value was misrepresented by over-sampling rural groups.
