# Supplementary material for: Factors Associated with Variations in Population HIV Prevalence across West Africa: Findings from an Ecological Analysis
Source: PLoS One. 2015 Dec 23;10(12):e0142601. doi: 10.1371/journal.pone.0142601 (PMC4689529; doi:10.1371/journal.pone.0142601)

S3 File - Figures on Anti-Retroviral treatment for countries across West Africa.

Table 1: Gives figures on the total number of individuals in each country who are receiving ART treatment. Figures for the total population size are estimated from The World Bank. HIV prevalence estimates are calculated by taking (total numbers HIV+/total population)

Countries	Total numbers HIV+	Total number of people receiving ART					World Bank estimates	2010-2014 estimates
		2008	2009	2010	2011	2012	Total population 2009-2013	HIV prevalence
**Benin**	123881	12078	15401	18230	19930	26035	10,323,474	1.2
**Burkina Faso**	169348	21103	26448	31543	36248	45910	16,934,839	1.0
**Cameroon**	956920	59960	76228	89455	105653	122783	22,253,959	4.3
**Côte d'Ivoire**	751695	51820	72011	75237	82721	110370	20,316,086	3.7
**Gambia**	29588	770	921	1869	2891	3571	1,849,285	1.6
**Ghana**	362664	21548	30265	40575	54589	69870	25,904,598	1.4
**Guinea**	199668	9212	14999	20430	23135	26666	11,745,189	1.7
**Guinea-Bissau**	90325	1832	2764	3632	5104	6101	1,704,255	5.3
**Liberia**	47234	2017	2970	4412	5839	5478	4,294,077	1.1
**Mali**	168318	16475	21100	24778	29237	28751	15,301,650	1.1
**Niger**	71325	2846	6445	7812	9420	11810	17,831,270	0.4
**Nigeria**	5902921	238659	302973	359181	432285	491021	173,615,345	3.4
**Senegal**	70666	9252	12249		12762	14692	14,133,280	0.5
**Sierra Leone**	91381	1950	3660	5552	8115	8259	6,092,075	1.5
**Togo**	170424	11211	16710	24635	29045	30311	6,816,982	2.5

Table 2: Estimates of total population size for those who are HIV+ for countries across West Africa beginning in 2008 and assuming a 3% annual growth rate.

Countries	Population size estimate based on World Bank estimates (3% growth rate)				
	Pop 2008	Pop 2009	Pop 2010	Pop 2011	Pop 2012
Benin	123882	127598	131426	135369	139430
Burkina Faso	174429	179662	185052	190603	196321
Cameroon	985628	1015197	1045653	1077022	1109333
Côte d'Ivoire	774246	797473	821398	846040	871421
Gambia	30476	31391	32332	33302	34301
Ghana	373544	384751	396293	408182	420427
Guinea	205658	211828	218183	224728	231470
Guinea-Bissau	93035	95826	98701	101662	104712
Liberia	48652	50111	51615	53163	54758
Mali	173368	178569	183926	189444	195127
Niger	73465	75669	77939	80277	82685
Nigeria	6080009	6262410	6450282	6643790	6843104
Senegal	72786	74970	77219	79536	81922
Sierra Leone	94123	96946	99855	102850	105936
Togo	175537	180803	186228	191814	197569

Table 3: Estimates for table 3 are calculated using the total number of individuals receiving ART for each respective year, divided by the total number of individuals who are estimated from table 2 and multiplied by 100 to obtain a percentage estimate.

Countries	Percentage of those HIV positive on treatment				
	ART 2008	ART 2009	ART 2010	ART 2011	ART 2012
Benin	9.7	12.1	13.9	14.7	18.7
Burkina Faso	12.5	14.7	17.0	19.0	23.4
Cameroon	6.3	7.5	8.6	9.8	11.1
Côte d'Ivoire	6.9	9.0	9.2	9.8	12.7
Gambia	2.6	2.9	5.8	8.7	10.4
Ghana	5.9	7.9	10.2	13.4	16.6
Guinea	4.6	7.1	9.4	10.3	11.5
Guinea-Bissau	2.0	2.9	3.7	5.0	5.8
Liberia	4.3	5.9	8.5	11.0	10.0
Mali	9.8	11.8	13.5	15.4	14.7
Niger	4.0	8.5	10.0	11.7	14.3
Nigeria	4.0	4.8	5.6	6.5	7.2
Senegal	13.1	16.3	-	16.0	17.9
Sierra Leone	2.1	3.8	5.6	7.9	7.8
Togo	6.6	9.2	13.2	15.1	15.3

Figure 1: Regression analysis showing relationship between HIV prevalence in different settings and % of individuals receiving ART

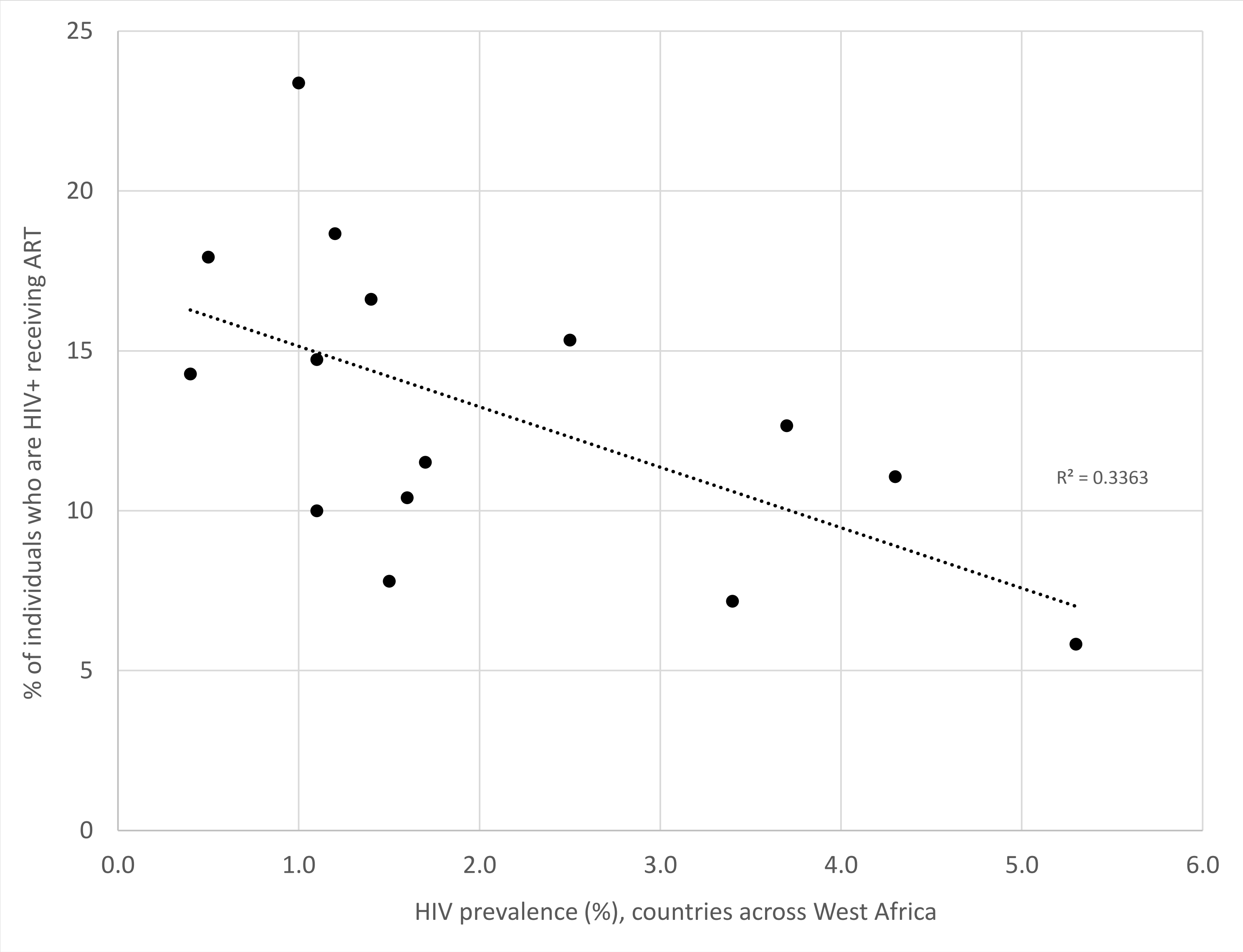

Supplement: S3 File — (PDF) [file pone.0142601.s003.pdf]
