# Supplementary material for: Factors Associated with Variations in Population HIV Prevalence across West Africa: Findings from an Ecological Analysis
Source: PLoS One. 2015 Dec 23;10(12):e0142601. doi: 10.1371/journal.pone.0142601 (PMC4689529; doi:10.1371/journal.pone.0142601)
Supplement: S4 File — (PDF) [file pone.0142601.s004.pdf]

S4 File - Age at sexual debut and association with percentage of males and females reporting 2+ partnerships in population.

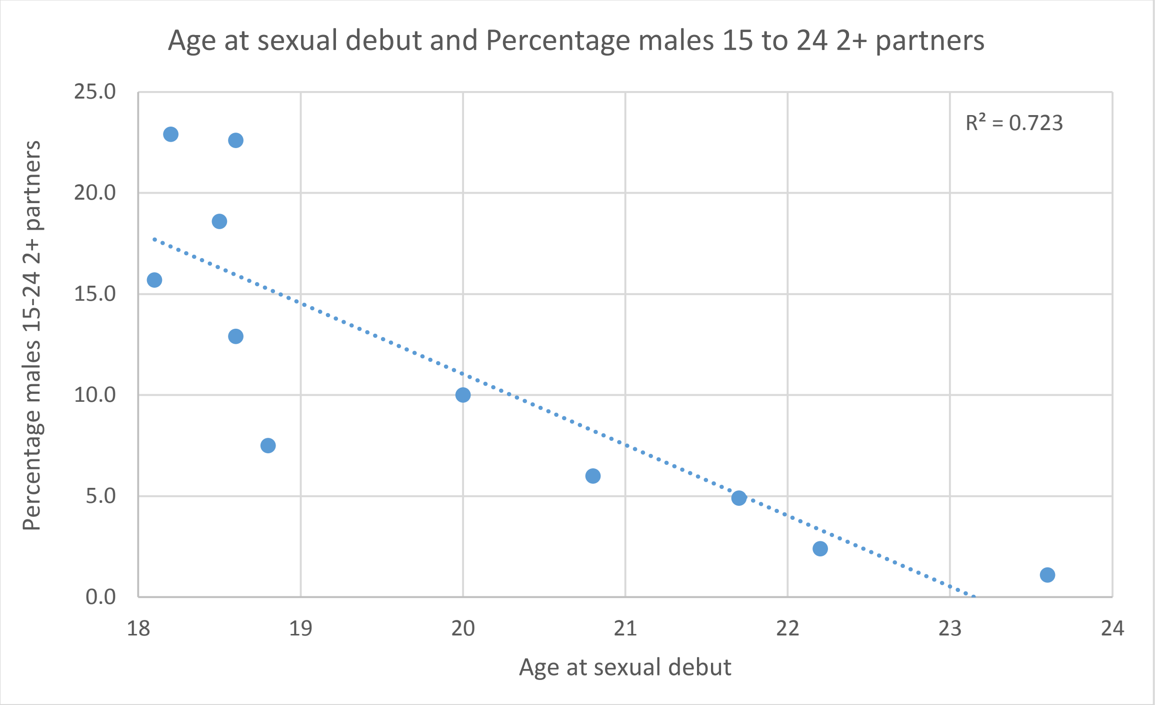

Age at sexual debut for males 15-24 and percentage of males aged 15-24 with 2+ partners p=0.001

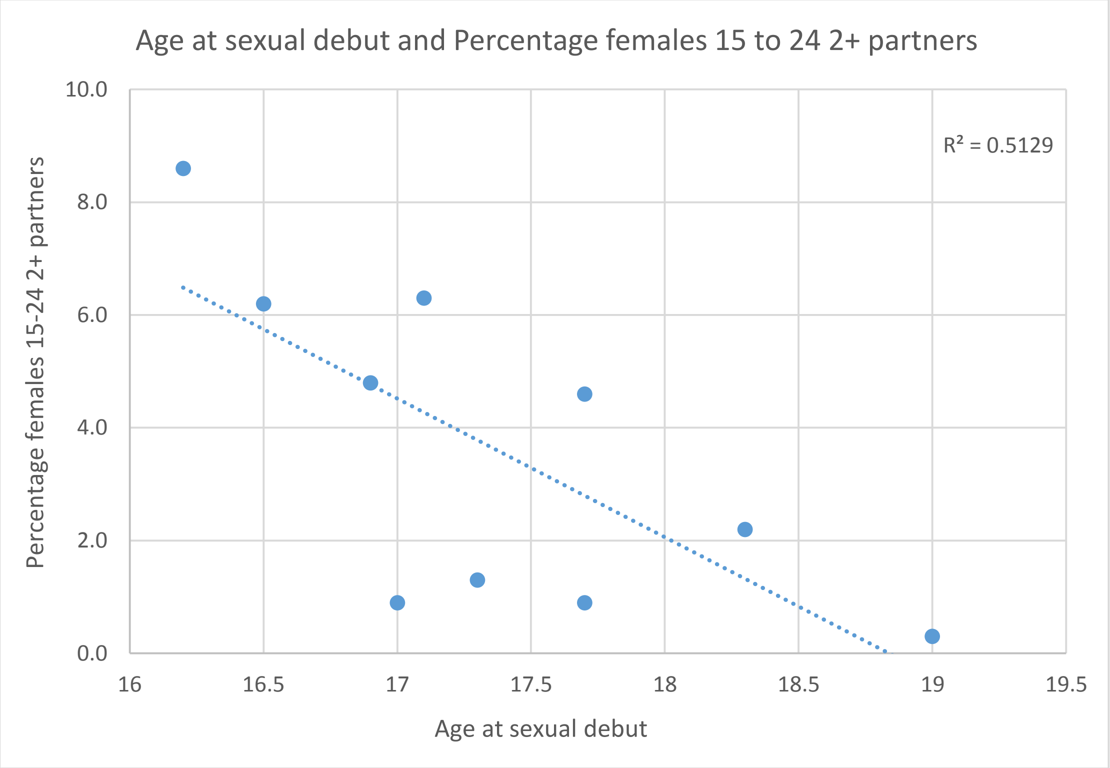

Age at sexual debut for females 15-24 and percentage of females aged 15-24 with 2+ partners p=0.03
